# Hospice care providers experiences of grappling with medical assistance in dying in a hospice setting: a qualitative descriptive study

**DOI:** 10.1186/s12904-021-00740-3

**Published:** 2021-04-12

**Authors:** Shannon Freeman, Davina Banner, Valerie Ward

**Affiliations:** 1 School of Nursing, University of Northern British Columbia, 3333 University Way, Prince George, British Columbia V2N 4Z9 Canada; 2 Northern Medical Program, University of Northern British Columbia, 3333 University Way, Prince George, British Columbia V2N 4Z9 Canada

**Keywords:** Medical assistance in dying, Advocacy, Physician assisted suicide, Euthanasia, Palliative nursing, Qualitative, Terminal care

## Abstract

**Background:**

Rapid implementation of Medical Assistance in Dying (MAiD) across care settings has challenged providers and organizations, including hospices, to develop and implement new modes of practice. The aim of this study was to examine the effects that legalization of MAiD has had on hospice care provider roles within the non-provider context.

**Methods:**

Eight in-depth semi-structured interviews were conducted and a qualitative descriptive approach used to examine hospice care providers experiences in a small western Canadian city. In the study context, patients who choose MAiD are cared for until immediately prior to the procedure when they are transferred off-site to undergo MAiD. Inductive and thematic analyses were undertaken.

**Results:**

Participants experienced practical, philosophical, and professional challenges. Despite the overwhelming desire to support patient autonomy and decision-making, some interpreted patient choice for MAiD as rejection of the natural death experience at the hospice. Patient choice for MAiD initiated a new and different pathway of end-of-life care. While participants felt uncertain how best to support patients undergoing MAiD, they shared mixed optimism on how their care provider roles were evolving as their level of experience broadened. While implementation of MAiD was rapid, the introduction of practical and professional supports has remained slow to materialize, leaving many providers to navigate their own personal and professional positions and practices.

**Conclusion:**

Care providers require a multi-faceted range of clinical, legal, and logistical supports at the practice, organizational, and health system levels, to facilitate care delivery to those requesting and undergoing MAiD and to promote coordinated and holistic patient-centered care. The different pathway for those who chose MAiD may lead care providers to struggle with relational challenges and interpersonal unease. Further research may address how to support those undergoing MAiD within the hospice context.

## Background

Hospices provide holistic comprehensive person-centered care to persons nearing death, as well as to those close to the individual nearing death including family and friends. Hospice care “is aimed at relieving suffering and improving the quality of life for persons who are living with, or dying from, advanced illness or are bereaved” [24]. In June 2016, the provision of Medical Assistance in Dying (MAiD), including access to euthanasia and assisted dying, was legalized in Canada [21]. National organizations sought to delineate MAiD and hospice palliative care noting that “provision of MAiD is a practice distinct from palliative care” [12]. While the introduction of MAiD has provided new options for those experiencing life limiting illness and irremediable suffering, questions about its fit within hospice and palliative care have persisted. The CHPCA emphasized that “Healthcare articles and the general media continue to conflate and thus misrepresent these two fundamentally different practices. MAiD is not part of hospice palliative care; it is not an “extension” of palliative care nor is it one of the tools “in the palliative care basket”; [4]. Since the legalization of MAiD in Canada, hospice care providers have been challenged to adapt and adjust. While some hospices have chosen to offer MAiD in their facilities, other facilities including faith-based organizations have declined based on conscience grounds [36]. For most facilities, full participation in MAiD is expected [36] and some hospices that have decided not to offer the provision of MAiD have faced significant challenges and pressure [25, 39]. While there is a growing body of literature on MAiD, few studies have captured the experiences of those delivering care within the non-provider hospice setting. Therefore, this study examines the experiences of care providers working within a non-MAiD provider in-patient hospice.

Residential in-patient hospices provide compassionate pain, symptom, and comfort care to persons at the end-of-life who may no longer be cared for in their home [38]. Hospice care is commonly provided by an interdisciplinary team involving a wide range of care providers including physicians, regulated nurses, care aides, social workers, and grief support counsellors as well as volunteers and administrative staff. In Canada, there are less than 100 residential in-patient hospices [10]. While eligibility criteria for hospice care may differ by facility, the majority who access hospice services require symptom management and care, have ceased active curative treatment, and have an estimated prognosis of less than 3 months [10, 11]. It is estimated that nearly two thirds of those who die each year in Canada may benefit from hospice and palliative care [8], however, less than 15% of Canadians with a need for palliative care are able to access services [7].

While Belgium, the Netherlands and some US states legally permit physicians to provide assistance in dying, Canada is the only jurisdiction where legislation permits MAiD to also be provided by nurse practitioners [3]. Under the Criminal Code of Canada, and in accordance with a list of specified criteria, only physicians or nurse practitioners are permitted to determine eligibility for MAiD and to provide MAiD by prescribing, providing, and/or administering a medication to cause the death of the individual [23]. Other regulated nurses (registered nurses, licensed practical nurses/practical nurses, and registered psychiatric nurses), as well as other care providers (e.g., grief counsellors, social workers, care aides, and volunteers), are not permitted to provide MAiD nor to determine eligibility [23]. Eligible persons for MAiD must qualify to receive federally or provincially funded health services, be mentally competent, at least 18 years of age, have a grievous or irremediable medical condition, voluntarily request to receive MAiD without external pressures or influences, and be capable to provide informed consent [21]. Eligible persons for MAiD must be mentally competent able to provide informed consent at time of choosing MAiD, as well as at the time they choose to undergo the MAiD procedure [21]. Advanced directives to permit MAiD at a future point in time are not permitted [27]. The MAiD procedure is conducted by an authorized physician or nurse practitioner who may prescribe and/or administer medication to intentionally result in the death of the patient who has deemed eligible to receive MAiD [6, 20]. While there is a 10-day waiting period required between the time that a request for MAiD is signed to the time when the MAiD procedure may be performed, exceptions may be permitted if the individuals death is ‘fast approaching’ or when there is a concern that the individual may lose capacity to provide informed consent [21].

Deaths from MAiD accounted for 2.0% of all deaths in Canada in 2019, with 5631 deaths from MAiD reported [23]. Approximately 67% of persons who received MAiD reported cancer as their underlying medical condition [23]. The mandatory 10-day waiting period between time of decision to MAiD procedure was shortened for 34.3% of patients who received MAiD [23], and for 58.4% of patients with an estimated prognosis of less than 1 month [16]. Furthermore, 15.2% of patients who requested MAiD died of natural causes before they were able to undergo MAiD [23].

Controversy surrounds the provision of MAiD within hospice care in Canada [1, 16]. Hospice palliative care organizations, including the International Association for Hospice Palliative Care, CHPCA, and the CSPCP, posit that provision of MAiD conflicts with the philosophy of hospice palliative care which does not seek to hasten death [9, 12, 15]. Debate surrounds conscientious objection from an organizational perspective and whether hospice societies may abstain from providing MAiD. Concerns about accessibility and availability of both MAiD and hospice care continue [18, 43]. These varied positions were illustrated in a recent study by Freeman [18], who found that less than half of participants in their study felt it was appropriate to conduct the MAiD procedure in a hospice setting.

There is growing and immediate need to understand how the role of hospice care providers has been impacted following the rapid implementation of MAiD, especially in the context of non-MAiD provider in-patient residential hospice facilities. As holistic end-of-life hospice care is typically provided by an interdisciplinary team, this research set out to enhance our understanding of the effects on non-MAiD hospice care providers role following legalization and rapid implementation of MAiD in the Canadian context. We sought to include those engaged in care delivery, including physicians, nurses, grief support workers, and volunteers. Here, we build upon our previous work examining the broad effects that introduction of MAiD has had on these care providers [41] to focus more narrowly on how the care provider experience caring for patients who chose to undergo MAiD effects their role as a hospice care provider.

## Methods

We undertook a qualitative description study [34]. Qualitative description is a flexible and pragmatic approach to research that allows for the discovery of human experience within the naturalistic context [33]. While interpretation is inherent to the qualitative approach, qualitative description enables the generation of rich account of experiences, while preserving a connection to the language and syntax of the participants [29, 33]. For the purposes of the study, this approach was selected due to its alignment with the study goals and its ability to assist in the rapid translation of findings into practice.

### Sample

Eight in-depth semi-structured interviews were conducted with hospice care staff. Convenience and purposive sampling techniques were utilized to recruit a diverse cohort of non-MAiD, hospice care providers. Prospective participants were identified and were recruited via e-mail and word of mouth. Participants were eligible to participate if they were a formal care provider (e.g., registered nurse, care aide, grief support worker, and care manager) or facility-affiliated informal care providers (e.g., trained hospice volunteer or program coordinator). All participants provided informed consent. Interviews were conducted in a confidential room on-site and lasted between 30 and 60 min. Interviews were audio recorded (with the exception of one participant) and field notes were taken. Recruitment continued until saturation was achieved, considered the point at which no new substantive information was generated [40].

### Study setting

The study was undertaken in a geographically-isolated medium sized city (population < 100,000) in western Canada. All participants were engaged in hospice care provision in an in-patient hospice facility which did not permit provision of MAiD procedures on site. In this study, care providers at the hospice care facility were not involved in the assessment nor in the provision of MAiD. Instead, if a patient inquiries about MAiD, the hospice care providers would refer the patient to a physician or nurse practitioner for further information and assessment if requested. As per facility policies, if a patient requests MAiD they may be cared for until the time immediately prior to the procedure at which time they must be transferred off-site to receive the procedure.

### Data analysis

Guided by the qualitative description approach, inductive and thematic analyses were undertaken. First, transcripts were cleaned and reviewed for accuracy and were then read closely by the team to gain familiarity with the data and to yield the early identification of important concepts. Following this, transcripts were uploaded into the NVIVO 12 and were coded and organized thematically [31]. Initial coding activities focused on labeling and grouping data. These were reviewed by two researchers independently (SF and DB) and areas of convergence were discussed until consensus was achieved. As codes became increasingly refined, team members worked to identify commonalities and differences, as well as to map the emerging patterns and themes.

To promote rigor, peer checking and prolonged engagement with the data was undertaken and rich descriptions were generated. Reflexive memos and regular analysis meetings allowed for the documentation and exploration of relevant presuppositions and reflections on the emerging data. The study received harmonized ethics approval from the University of Northern British Columbia and University of British Columbia (#E2017.0518.038H). Written informed consent was received from all participants.

## Results

Following analysis, three themes emerged, including Reconciling patient choice for MAiD and termination of hospice care, Relational challenges and interpersonal unease, and Initial steps to normalizing their response to MAiD. Overwhelmingly, care providers worked to foster patient-centered care while navigating practice, professional, and regulatory challenges.

### Patient choice for MAiD and the termination of hospice care

The introduction of MAiD gave rise to philosophical and practical shifts that impacted participants self-perceived role to provide person-centered care. Participants described how MAiD changed and challenged their understanding of the nature of death and dying within the hospice setting, as well as their experience of delivering care. Participants described MAiD as a different mode of dying, characterized by a sudden and rapid transition to death with a specified and predetermined trajectory. As one participant explained, *“MAID is a sudden end to death … MAID is a more determined time date where life will end” (P1).*

When reflecting upon their experiences and perspectives, participants frequently talked about the timing of MAiD within the patient’s illness journey. They identified that patients undergoing MAiD were typically not at advanced stages of their disease, or were not as close to death, as what they normally expect to see when caring for patients experiencing a natural death within the hospice setting. As one participant explained *“They are still doing quite well, it is hard for me to work in this field, it still feels like they have months in them, it is hard like why wouldn’t you want to live as long as you can type thing” (P1).*

When recalling these experiences, many participants reflected upon the patients’ condition immediately prior to MAiD. Through this, they explained how they struggled to make sense of the patients’ imminent death within the context of their illness trajectory and daily functioning. One participant reflected, “[*the patient] got up every morning, did [their] hair, did [their] makeup, [they] really enjoyed, [they were] on a high percentage of life when [they] passed, which as a caregiver still surprises me” (P2).* While another participant reflected that another patient was:

*not really showing any of the symptoms that we are used to dealing with. I think the only symptom was really just anxiety and loss of control … [they were] quite well, quite fit, and [they] just walked to [their] car and left for the procedure. It was pretty harsh and stuff but again the care was great, everybody cared for [them] up until the end. But I think from my perspective, that even as it seemed so soon, [they] seemed to pre-empt everything. (P3).*

These experiences with patients who underwent MAiD challenged participants’ perceptions of what a dying person should look like. They described how the patients who chose MAiD did not align with their expectations or image of a patient who may need MAiD. This mismatch between expectation and reality was especially disconcerting for some care providers to reconcile. Care providers posited that some patients may request MAiD out of fear of suffering but want the option just in case:

*I believe that for a lot of other people it is a peace of mind where they know they have the option because even though with medicine with the way that we have it, there is still a huge fear that people feeling they will die suffering and so even though they don’t use it, it is good for them to know that it is available to them. (P1).*

In response, many care providers sought to make sense of this journey, often grasping for explanations or questioning the unknown gaps.

Participants struggled to reconcile the provision of MAiD within hospice care, both in terms of its general fit within the scope of hospice care, as well as within the physical context of an in-patient hospice facility that cares for patients awaiting MAiD but where MAiD is not provided. Participants recognized that MAiD interrupted their expectations and perceived ideas about the temporal sequence of the patient care journey within the hospice context. This was seen to change the dynamic of care. When speaking about their practice, participants commonly described the experience of a natural death as a journey. They recognized that while the death would be the end point for most patients within the hospice setting, MAiD disrupted the normal patterns of care.

*They might not be a person that would normally pass away quick. It would be someone who is seriously speeding up their death. Which is interesting because it takes the natural process out of there. It sometimes can make it so that the person is not dealing with death. (P2).*

For many, this led to conflicting views on how MAiD and hospice care provision fit philosophically and practically, particularly within the in-patient hospice context where MAiD is not provided. This gave rise to diverse, and sometime conflicting, views amongst interdisciplinary care team members. Some of the participants commented:

*I am not sure that hospice is where MAID should be done but certainly hospice can play a role in supporting families and people as they make those decisions … I think that the philosophy of hospice has always been that we do not prolong or hasten death. To me that seems gentler, but the other piece, the more practical piece is that it is a really polarizing conversations. (P5).*

*Palliative care is to let the person die at the end of their life the way that they want to which is where hospice comes in to give them that option, so MAID being allowed to be part of that option should be within hospice, should be within the guests’ right at hospice, should be part of that option … knowing when they will die so they can live the last days of their life the way they want to, knowing that they are not going to be in pain, that they are not going to be in suffering, then it should absolutely be an option for them. (P6).*

Variations in perspectives on philosophical alignment and the conceptual fit between patient choice for MAiD and provision of hospice care seemed interwoven with their professional comfort or discomfort with transition of the patient to an external location outside the in-patient hospice facility to undergo MAiD. In this study, patients who chose MAiD would be transferred to an external site to receive the procedure from a different care team. This transition ignited controversy among care providers when aligning whether or not this was acceptable care and fit into best-practice end of life care, as well as diverse views around the optimal place and space for MAiD. One participant noted that MAiD should be available “*wherever somebody wishes*” (*P1*), while another commented that: “*I prefer that patients don’t have to be moved … hospice is a place to come for comfort, and for the best possible way to leave this world for the patients and their families, it should be about patient choice” (P4).* Likewise, another explained that:

*In the patient’s home – that’s the ideal. Because the people that I have seen that have chosen MAID, they are very clear on what they want, there’s no real hesitation, it’s quite remarkable actually. I think when they are in an environment where they are surrounded by people who love them, and the people who care for them, I think that would be the best way for the person who is dying, but also for the family members afterwards, for the grieving process. (P5).*

While most reflections about MAiD focused on the conceptual or practical fit in hospice care, one participant shared concerns about how MAiD might impact access to already finite and limited hospice resources. In particular, they were concerned that by allowing persons who request MAiD to spend the mandated waiting period within hospice, they may block access for others requiring hospice palliative care. They explained:

*If someone is here waiting for MAID, and someone who is palliative and dying and wants to be here … how do we utilize beds now, is it ok to tie up beds for 10 days now? do we still want to wait and somebody dies in hospital while waiting for a bed. (P1).*

### Relational challenges and interpersonal unease

When reflecting upon their experiences of delivering hospice care, participants explained how MAiD changed the nature of care they were able to provide and connections they created with patients. For some, a patient’s decision to undergo MAiD left them contemplating the balance of supporting the patient’s autonomy with their own understandings of high-quality patient-centred hospice care. Furthermore, the transition to MAiD left them questioning the success of their care or whether participants had the chance to experience the spectrum of care available. One participant commented:

*It seems like a conflict of interest because if we are the ones who are also able to, or potentially, promote it or something, it’s kind of like saying that we aren’t able to do our job well enough, like we can’t control your symptoms so here why don’t you just end it. So, in one sense, it is kind of looked at a little bit like a failure on our part to provide good enough symptom control that people would choose to go ahead with MAiD. So, I do think it would be a conflict to have MAiD at hospice. (P7).*

For others, there was concern that by undergoing MAiD the patient was missing out on the ability to experience the natural dying process and the full spectrum of hospice care provision. For these participants, there was a sense that:

*It’s sad because palliative care is such a baby in evolution, it’s just evolving right now … if we do good palliative care right now, would it change [the decision for MAiD], there is still a lot of people suffering. People don’t prescribe adequately. People don’t treat symptoms adequately, because it should be it can be wonderful and if we can bring palliative care into homes where we can support, and it is a tremendous stretch in one’s life to add depth and growth to humans and brings families back together. (P1).*

While also challenging their perceptions of hospice palliative care, participants explained how the initiation of MAiD gave rise to a distinct separation in care delivery as they were not permitted to fully engage in conversations with patients about MAiD due to legal and policy requirements. During the early stages of MAiD assessment, many care providers are excluded from engaging in direct communication and consultation about MAiD, which gives rise to a disconnection and fragmentation of the patient-provider relationship. Participants reflected that this gap in communication left them unaware of the patients motivations for MAiD and unclear about what education and supports had been provided or were needed. Some participants commented:

*We are not sure what is said, so during that admission and the assessment [for MAiD], is the full scope of what we do fully presented? Have they discussed fear? What is their biggest fear? Is their fear breathing, pain, have they discussed what palliative care and hospice care can do to support that and we don’t know that between the physician and patient. (P3).*

*Because it is so new, we get asked all the time, as a nurse right now, I don’t have the authority to discuss it other than to direct the person to their GP who then would consult with the person and consult with family and initiate the procedure, but people have questions and they usually come to the nurse to ask, and so you kind of feel you are neglecting them by not answering them, because we answer all other questions but there we have to tell them to talk to the doctor about it. And it is difficult to know how they perceive it, they may perceive it that you don’t want to have anything to do with it, that you feel that they shouldn’t be doing it, all kinds of questions come up … I feel that I disappoint clients by not being able to tell them or answering their questions. I am just truthful that legally those are questions that have to be directed to a GP. (P1).*

In light of this separation, some participants described how they were often left on their own to make sense of the patients decision-making and motivations to choose MAiD. These feelings of uncertainty were amplified by the requirement that the patient transfer out of hospice to receive the MAiD procedure from different care providers in a different care setting. The transfer out of the facility was seen as an abrupt termination of the patient-provider relationship during a time in which relational care was perceived to be most valuable. It was at this point that many participants felt most conflicted about MAiD. At this point, further separation occurred whereby the participants were not able to be present for the death from MAiD as they usually would be during a natural death in the hospice facility. On a psycho-emotional level, participants experienced sadness as they were unable to provide ongoing emotional care for the patients and the patients’ family and friends. For some, this gave rise to feelings of inadequacy and rejection, as they were not able to provide full person-centred care, as well as communication challenges as they navigated the awkwardness and discomfort of the final farewell and greetings. For many, the discussion of MAiD as an end to their journey of life culminating in a chosen, expected, and scheduled time of death “doesn’t fit in our usual conversation”, leaving many not knowing what to say or lacking the experience and language to navigate these difficult conversations.

*For 2 days before their death, I googled high and low, I googled trying to find out what to say and, in the end, I decided that I was just going to leave a card to say my goodbyes … I didn’t know how to terminate my relationship, what do you say to people ‘have a good death’? so I fretted a good 2 days not knowing what to say, I consulted with all of my peers about ‘what do I say to the person?’ and at the end they brought it up herself, and I was greatly thankful that it was just a goodbye, but I was also truthful to them and saying thank you for bringing it up because I was at a loss of what to say to you, and it affected us a great deal, all of us, it was just something we have never done, and to watch family every day the day came closer their faces withdrew deeper, until it was almost ghostly scenario, just broke our hearts, but later on family said it was the right thing for them to do, and it worked for them, but it was really really tough, and it really affected us. (P1).*

For some participants, observations of the reactions of family members, along with their own experiences of providing care, demonstrated the challenging of knowing what to do and say. Participants emphasized the importance to demystify the language to describe death and dying from MAiD.

*I think as a community we need to talk more about death. It’s not scary, I think people have ideas in their head about who is having MAID, they are picturing the emaciated person withering in pain having MAID, that’s not who is having it. I think if people know that there is a beauty in dying, these people are not afraid of death, they are going openly to their death, they are afraid of dying, so we need to demystify dying (P3).*

This discomfort with the professional role rules that they are not included in the assessment and decision-making process for MAiD, along with the limited role in it provision, limited their abilities to fully engage in conversations around MAiD. Participants worked to provide support to patients and their families as best they could during these final moments prior to transfer and further recalled the diverse range of emotions and responses family members may experience. Two participants explained:

*Avoidance, no just avoidance of the topic, but to see the family so withdrawn and so stoic I still have the facial expression of the [relative] in my mind of how [they] walked around that last night it was tough to see, so definitely not looking that way, looking busy, not that I did not go in and do my checks, but I definitely felt more uncomfortable doing it and it was hard not to burst out crying, because the thought was just mortifying that tomorrow at whatever time you are going to be dead. (P1).*

*Last experience was with a [patient] who kept the decision secret from [their] family until the day that it was being performed. And [the patient was] very ok with the decision [themself]. I know there was lots of high emotions between the staff and the family when they found out because they were all there in the morning (P6).*

During the final period prior to MAiD, participants reflected upon the awkwardness of bridging normal daily activities with the imminent transfer for MAiD. One participant recalled helping a patient prepare to leave, commenting that:

*[they] asked me to take [their] cellphone, just clothes and shoes, what do you really need, [they] didn’t need [their] purse [their] wallet, where [they] was going, [they] had nothing …, surreal, I was going to wear this color, so stupid the convo, try to be cheery, guests, staff put on a brave face, hold it together until they’re gone as soon as the door closes, everybody feels ok to not be ok. (P4).*

As well as observing the grief of others, participants described their own experiences with grief and loss associated with the patient’s choice to receive MAiD, including grieving their inability to do their job fully and the anticipatory grief knowing that the patient would die shortly after they transferred from the hospice. For some, this grief emerged from a perceived rejection of the hospice care they provided to support a natural death in their in-patient hospice facility. One participant explained:

*When [they] died, I bawled, it rocked me to the core, that happens every now and then, even when you have good boundaries of empathy. Why did this really bother me? It is because there was something in that grief that you see in yourself, or there is a piece that somehow relates to you. For me, there was something that did not sit well with me, what I saw was that my personal belief, although I don’t reserve judgment, and I don’t judge in any death situations, I just value life a lot and I think that [their] family had a hard time with it. (P2).*

### Normalizing their response to MAiD

The emotionally charged realities of caring for a patient who chose MAiD required participants to be conscious of how they care for the patient and their families and in how they provide self-care and access supports for themselves. Existing in-facility care supports continued and included debriefing and informal supports from managers and others. In the absence of self-care training specific to MAiD, participants described a variety of self-care strategies they had adapted to help cope with their experiences. This included personal coping strategies (breathing, exercise, talking to others), as well as adapted facility strategies (lighting of candles and flexibility in work expectations). In one case, a participant took time off following the patients transfer to undergo MAiD, while others focused on keeping busy by immersing more deeply in care activities for other patients and their families.

It’s always shocking. I feel like as time goes on it will become more normal but at this point for me personally, it’s always shocking, it’s kind of a physiological response. And I need to concentrate on breathing and coming back into my body so I can be present for the people that might want to talk to me. (P5).

While a multitude of emotions were experienced, some participants recognized that they were getting more accustomed to the process and were gaining greater comfort with MAiD as their experiences grew. For some, MAiD was “*becoming more normal” (5),* while others noted that the intensity of the feelings were subsiding over time but maintained that the raw emotional response remained. Others recognized that communication became easier over time and that they were becoming more confident in caring for the person awaiting the MAiD procedure. Yet, there was discomfort and fear in becoming too complacent and desensitized as MAiD became more familiar. As one participant described:

*other than the initial one, where there is avoidance, socially, it was harder and harder to communicate, but that was the first time and it’s as the not knowing and its odd. … All of that has already become more acceptable. It was less of a shock, it was easier to say goodbye, and it’s kind of frightened me. It frightened me how easily I have adjusted, to this in a short period of time, and it worries me are we not taking death seriously enough anymore, trying hard enough if that is now an option. And I became very conflicted over how little it bothered me to have this person die with MAiD and go ahead with MAiD and I said goodbye and I carried on working, and I felt guilty and a little bit desensitized, and I was a little distressed by that now. (P1).*

Another care provider described how they have adopted a coping strategy to prematurely disconnect from fostering a closer patient-provider relationship. “*I have disconnected a little bit, it’s not about being rude, but I need break to not get as involved, I am still friendly say good morning, maybe I don’t want to get to know them as well, to save myself a little.” (P4)* This disconnect from the patient after they chose MAiD was perceived as a self-care strategy to protect themselves from the raw emotional feelings they had experienced with previous patients.

## Discussion

While assisted dying has been available in several countries across the world, the introduction of MAiD in Canada represents a new and evolving process. Consistent with its rapid introduction, healthcare organizations are developing new modes of practice and there is ongoing interest in how MAiD is experienced and enacted across environments. Despite a surge in studies exploring MAiD, few studies have tackled how MAiD is experienced within hospice and non-provider of MAiD settings. Moreover, MAiD within the context of hospice palliative care has remained a highly contentious and widely discussed issue [12, 36]. In this study, the polarizing nature of MAiD, along with the practical, philosophical, and professional challenges that emerge within a non-provider of MAiD setting, were uncovered.

While reflecting upon the experiences of MAiD within an in-patient hospice care environment where the MAiD procedure is not conducted, participants highlighted how patients undergoing MAiD divert to a new pathway towards death. Referred to as ‘different’, ‘artificial’, ‘non-natural’, and ‘planned’, participants made a clear distinction between the end-of-life journey and the hastened and scheduled timing of the death for those who chose MAiD compared to patients who did not. Through this process, participants recognized the introduction of a procedure with the explicit aim to hasten death as a departure from usual practice within the hospice setting where patients receive care to alleviate suffering and improve quality of life across the natural lifespan.

Care providers worked to navigate the unchartered territory alongside patients on the MAiD trajectory of death within a non-provider of MAiD hospice in-patient care setting. They worked to remedy the moral complexities and philosophical and practical uncertainties of MAiD within their work and care setting, while attempting to provide high quality patient-centered care. While all participants strongly reiterated their desire to support patient autonomy and decision-making, participants recognized that the request for information about MAiD initiated a disrupted, distinct, and fragmented care pathway, a pathway that excluded most frontline hospice care providers. This separation occurred in part due to the governing Canadian legislation which constrains and limits the roles of some healthcare providers, such as registered nurses [3] but also, because the teams supporting the MAiD process are typically different from those delivering direct patient care and that the provision of MAiD requires a late and abrupt transition away from hospice care. Additionally, we have previously reported the challenges hospice care providers face to reconcile their anticipated/expected vision of whom may request MAiD with their first-hand observations of which patients who chose to undergo MAiD [41]. Moreover, we have also previously recognized the conflict some care providers experience when observing exceptions to the 10-day waiting period due to potential that the person would die naturally before being able to receive MAiD and/or fear that the person requesting MAiD may not be capable to consent following the full 10-day period [41].

In Canada, at time of this study, consultation and assessment for MAiD, for patients in the hospice facility, were provided by physicians and/or nurse practitioners not based at the hospice care facility. Hospice care staff were not involved in conversations specific to the decision-making process for patients choosing MAiD from point of assessment through the reflection period to point of transfer out of the facility for MAiD, leading to a disconnect in patient – care provider communication. For many frontline care providers, including registered nurses, nurse managers, and volunteers, this exclusion from conversations about MAiD left them feeling awkward and unclear on how best to collaborate with the patient, family, and wider care team. Conversations and requests for assistance in death are commonly experienced by healthcare providers, including nurses, and are well documented across a diverse range of clinical settings [17, 22, 35]. For example, approximately 1 in 5 hospice volunteers reported that a patient had initiated discussions about assisted death [14], while studies of nurses demonstrate the frequent and often morally challenging interactions that exist with patients requesting assistance to end their life [26]. When reflecting upon the evolving separation that occurs upon a request for MAiD within non-providing hospice contexts, some participants perceived this as a failure or rejection of hospice care provision due to their inability to fully support the patients’ needs. Likewise, exclusion further decreased transparency in discussions and led some care providers to question whether the patient was aware of, and had access to, the full range of holistic care supports.

Previous research has shown that feelings of rejection and uncertainty around assisted dying can give rise to emotional and moral distress [18]. Here, accounts of moral distress were common, as care providers attempted to reconcile MAiD within the context of care and hospice environment. Some care providers were able to adjust to a new focus of care, while others struggled with the inability to fully provide holistic hospice care and disconnected emotionally as a result. Disconnections and distress increased as the patient neared the time to transfer to another facility to undergo MAiD. While care providers overwhelmingly desired to promote patient autonomy, dignity, and respect, many were left unsure of how best to care for persons nearing the end of their hospice journey. For some, a lack of certainty around language made interactions with patients and those around them difficult. For participants in this study, a lack of clarity about what care was wanted, along with challenges in terminating the patient-provider relationship, led to some discomfort during those final days and hours. This was a stark contrast to the usual nature of care within the hospice setting and their presence during and after death.

The experience of navigating MAiD within a non-provider context challenged care providers to rethink and redefine their roles as holistic hospice care providers, leaving many unsure about how to best engage with patients undergoing MAiD and those around them. This led to diverse perspectives around the philosophical fit of MAiD within the hospice setting. Similar concerns around the philosophical congruence of MAiD within the hospice setting has been previously described, and the CHCPA defines three foundational concepts upon which hospice is based, including effective communication, effective group function, and ability to facilitate change [8], all of which become challenged within the context of MAiD. While these experiences were vivid and highlighted the dissonance between the desire to provide high quality patient-centered holistic care and the fragmented journey of MAiD, participants recognized that they were gaining increasing confidence and comfort as their skills and experience grew.

The rapid introduction of MAiD left organizations to implement services with limited direction or guidance. This filtered down to those providing frontline care, many whom were left to navigate their own belief systems and professional practices without effective supports, particularly with respect to nurses [30], physicians [37, 42] and volunteers [13]. As Antonucci and colleagues noted “there was an intense emotional toll taken on themselves [volunteers] and other health-care workers and their respective Ministry of Health was not providing enough support” [2]. Previous studies describe that while MAiD is becoming more common and expected, nurses may experience moral distress when a patient requests MAiD [32]. Similar concerns about a lack of guidance, support, and training have been raised elsewhere, including Garros’ accounts of MAiD legalization that highlighted the different nature of MAiD to other common practices at the end of life, including withdrawal of life preserving treatments or the initiation of palliative sedation, in addition to the lack of professional preparation [19]. Ongoing attention to the language and syntax of MAiD provides an opportunity to further deconstruct the experience of MAiD and to develop evidence-based supports that can aid the optimal care of patients undergoing MAiD and those around them. Furthermore, while discussions with care providers regarding palliative care and mental health supports have not been mandated, Downar [16] noted the utility of palliative care consultations to ensure patients are aware of the existing pain and symptom management options and hospice palliative care supports available. Further, Downar [16] stressed that mandating mental health consultation may also be useful to ensure capacity for patient informed decision-making. While this may reduce distress among care providers not engaged in the conversation, this can be challenging due to a growing shortage of specialists. Overall, formal supports remain limited and a lack of guidance at systems level has been previously documented. In the absence of formal supports, care providers began to develop their own systems of support and self-care including initiating informed debriefing or engaging in self-care activities. However, without supports to address the practical and structural challenges around MAiD, it is unclear if these uncertainties will ever fully resolve and what impact this may have on hospice care providers in the long term.

While MAiD presents eligible patients a new option through which to control and manage their death trajectory, the potential positive benefits that persons undergoing MAiD may experience may not be fully realized in the context of non-provider care settings, whereby disconnections in the delivery of care and practical, philosophical, and professional uncertainties exist. As Mills and colleagues [28] noted, there is a need for strong leadership to assist those navigating MAiD, as well as needs for practical supports for those caring for persons undergoing MAiD. Such gaps are heightened in the context of non-provider settings and this paper has begun to shine a light on the complexity of delivering holistic hospice care once MAiD planning is initiated.

MAiD represents a new and emerging practice within Canada. Following its rapid introduction, healthcare organizations, including hospices, were challenged with the need to develop policies and procedures to support its implementation. While this research has many strengths, some limitations exist. First, this research explores the experiences of care providers within a single non-provider hospice setting and further research to examine how MAiD is experienced across a spectrum of care settings would be valuable. Second, our study sample is relatively small. While saturation was achieved, it is possible that the inclusion of perspectives from a more diverse range of hospice providers may yield additional insights. Finally, experiences and practices of MAiD are evolving rapidly. Further longitudinal mixed methods research may be valuable in fully understanding the trajectory of this newly legalized practice in Canada, as well as further research to develop and implement evidence-based supports for interdisciplinary care teams.

Care providers require a multi-faceted range of resources, including clinical, legal, and logistical supports at the practice, organizational, and health system levels, to facilitate the delivery of high-quality care to those requesting and undergoing MAiD and to promote the delivery of coordinated and holistic patient-centered care. Resource development should focus not only on equipping care providers with tools specifically tailored to providing quality support for persons requesting MAiD, but also to provide support to the care providers themselves through education and self-care resources. Communication tools to support discussion, which also clarify the boundaries of the care provider-patient relationship in regards to MAiD, may help bridge some of the disconnect in the patient-provider communication. Care providers need a safe temporal and physical space to reflect on and connect with others about their thoughts, perspectives, and experiences as related to their understanding and experiences with MAiD. Safe environments that support discussion of polarizing topics, including moral distress, religious beliefs, and conscientious objection, are necessary. This includes opportunity to check in with members of the interdisciplinary care team for peer support and sensemaking of their ongoing experiences caring for patients requesting MAiD through talking, listening, hearing, and supporting themselves and each other [5].

## Conclusion

The introduction of MAiD in Canada represents a new and evolving choice for end-of-life care which has affected the dynamic of care within the hospice environment. The experience of navigating MAiD within a non-provider context challenged care providers to rethink and redefine their roles and left some uncertain about how best to support their patients and others. Care providers worked to navigate the unchartered territory of the MAiD trajectory within a non-provider hospice setting and sought to remedy the moral complexities, philosophical fit, and practical challenges of MAiD, while focusing on provision high quality patient-centered care. The initiation and provision of MAiD gave rise to a disrupted, distinct, and fragmented care pathway, one that excluded most of those at the frontline of hospice care. The different pathway for those who chose MAiD may lead some care providers to struggle with relational challenges and interpersonal unease. To deliver high quality palliative end-of-life care, framed through a patient and family approach and emphasizing whole person care in the hospice care environment, each member of the interdisciplinary care team needs to be able to access necessary education and supports. Therefore, a multi-faceted range of clinical, regulatory, and logistical supports at the practice, organizational, and health system levels are needed to specifically address care providers role since introduction of MAiD. Further research may also be warranted to address how these supports impact care providers within the hospice context.

## Data Availability

Not applicable.

## Abbreviations

CHPCA: Canadian Hospice Palliative Care Association
CSPCP: Canadian Society of Palliative Care Physicians
MAiD: Medical Assistance in Dying
